# IEDDA: An Attractive Bioorthogonal Reaction for Biomedical Applications

**DOI:** 10.3390/molecules26154640

**Published:** 2021-07-30

**Authors:** Maryana Handula, Kuo-Ting Chen, Yann Seimbille

**Affiliations:** 1 Department of Radiology and Nuclear Medicine, Erasmus MC, University Medical Center Rotterdam, Wytemaweg 80, 3015 CN Rotterdam, The Netherlands; m.handula@erasmusmc.nl; 2 Department of Chemistry, National Dong Hwa University, Shoufeng, Hualien 974301, Taiwan; ktchen26@gms.ndhu.edu.tw; 3 Life Sciences Division, TRIUMF, 4004 Wesbrook Mall, Vancouver, BC V6T 2A3, Canada

**Keywords:** pretargeting, click chemistry, bioorthogonal reaction, IEDDA, tetrazine, *trans*-cyclooctene

## Abstract

The pretargeting strategy has recently emerged in order to overcome the limitations of direct targeting, mainly in the field of radioimmunotherapy (RIT). This strategy is directly dependent on chemical reactions, namely bioorthogonal reactions, which have been developed for their ability to occur under physiological conditions. The Staudinger ligation, the copper catalyzed azide-alkyne cycloaddition (CuAAC) and the strain-promoted [3 + 2] azide–alkyne cycloaddition (SPAAC) were the first bioorthogonal reactions introduced in the literature. However, due to their incomplete biocompatibility and slow kinetics, the inverse-electron demand Diels-Alder (IEDDA) reaction was advanced in 2008 by Blackman et al. as an optimal bioorthogonal reaction. The IEDDA is the fastest bioorthogonal reaction known so far. Its biocompatibility and ideal kinetics are very appealing for pretargeting applications. The use of a *trans*-cyclooctene (TCO) and a tetrazine (Tz) in the reaction encouraged researchers to study them deeply. It was found that both reagents are sensitive to acidic or basic conditions. Furthermore, TCO is photosensitive and can be isomerized to its *cis*-conformation via a radical catalyzed reaction. Unfortunately, the *cis*-conformer is significantly less reactive toward tetrazine than the *trans*-conformation. Therefore, extensive research has been carried out to optimize both click reagents and to employ the IEDDA bioorthogonal reaction in biomedical applications.

## 1. The Emergence of the Pretargeting Approach

### 1.1. Limitations of Direct Targeting

Radiolabeled antibodies have been used over the last decades in radioimmunodiagnosis (RID) and radioimmunotherapy (RIT) to image and treat tumors. They are attractive biovectors due to their ability to target specific antigens expressed at the surface of tumor cells. Antibodies were first labeled by covalent incorporation of radioiodine to the tyrosine residues of the antibody [1]. Then, various radiometals were conjugated to the antibody via chelators [2,3]. The biological half-life of antibodies is typically expressed in days and, therefore, long-lived radionuclides such as indium-111 (*t*_1⁄2_ = 2.8 days), zirconium-89 (*t*_1⁄2_ = 3.3 days) and iodine-124 (*t*_1⁄2_ = 4.2 days), are required to warrant optimal accumulation of the radionuclide at the target site [1,4,5,6]. However, the long circulation of radiolabeled antibodies in the blood stream is a major challenge since it directly leads to unnecessary radiation exposure to healthy tissues such as the radiosensitive bone marrow [5,7]. Thus, many investigations on antibody fragments, active removal of labeled antibodies from the blood and pretargeting have been recently carried out to reduce radiotoxicity associated with radiolabeled antibodies [4,8,9,10].

### 1.2. Basic Principle of the Pretargeting Approach

**Figure 1:**
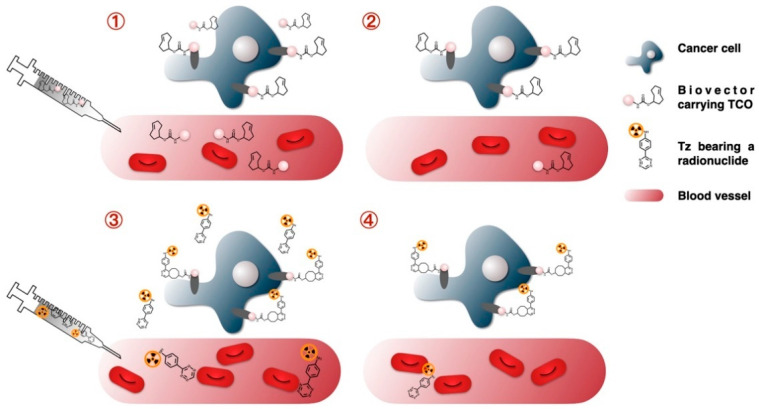
Illustration of the four basic steps of the pretargeting approach.

Pretargeting was introduced in the 1980s by Dayton D. Reardan and David Goodwin [11,12,13]. Many pretargeting strategies have been described in the literature, but methods based on biotin and avidin and bispecific monoclonal antibodies (mAbs) are the most common [5]. The pretargeting concept was developed to overcome the limitations encountered during direct targeting of tumors with radiolabeled antibodies [14]. It relies on four main steps (Figure 1) [7,15,16,17]. The first step consists of the administration of an unlabeled and modified biovector (i.e., monoclonal antibody) possessing the ability to bind an antigen or receptor and a radiolabeled small molecule. The second step is the slow accumulation of the biovector at the tumor site and its clearance from the body. The third step is the injection of the radiolabeled small molecule. Finally, the last step is based on the rapid binding of the radiolabeled small molecule to the biovector at the tumor site and its rapid clearance from the blood [10,18,19,20,21]. A clearing agent can also be employed at the end of the second step to remove the excess of biovector from the blood stream. The biovector is then transported to the liver or the spleen where it can be metabolized and eliminated from the body [14]. Several studies demonstrated that the clearing agent does not interact with the biovector already bound at the surface of the cancer cells but only with free molecules present in the blood circulation, as illustrated by SPECT/CT imaging studies [14,22,23,24,25,26]. Recently, Myrhammar and coworkers proposed a lactosaminated peptide nucleic acid-antibody conjugate as a potential clearing agent, whereas Cheal et al. developed a glycodendrimer-based clearing agent for pretargeting studies [27,28].

The main key factor of the pretargeting approach is the rapid pharmacokinetics of the radiolabeled small molecule to favor high radioactivity accumulation at the tumor sites while avoiding radiocytotoxic effects on healthy organs. However, success of the pretargeting strategy is directly dependent on the chemical reaction that allows efficient and selective binding of the radiolabeled small molecule to the biovector. Conventional bioconjugation reactions involving amine-carboxylic acid and thiol-maleimide result in undesirable conjugates in physiological conditions due to interferences with biomolecules present in the system. Hence, bioorthogonal reactions have gained a lot of attention for in vivo applications [29,30,31,32,33]. A bioorthogonal reaction is defined by: (1) fast reaction rate, (2) chemoselectivity, (3) no interference with the living system, (4) no toxicity and (5) stable starting materials and end-products. Moreover, the reaction has to occur in aqueous media at very low concentrations and under physiological temperature and pH [31,34,35].

## 2. Bioorthogonal Reactions

Click reactions were introduced in the early 2000s by Sharpless and coworkers [36]. According to the authors, a click reaction must be modular, stereospecific, high yielding, wide in scope and produce inoffensive products. However, the reaction should preferably be insensitive to oxygen and water and to occur in mild reaction conditions. Besides, the starting materials should be easily accessible, the products easily isolated and stable under physiological conditions. Importantly, a click reaction usually leads to a single product. To meet all these criteria, a click reaction needs to have a very high thermodynamic driving force, usually greater than 20 Kcal·mol^−1^ [36]. A bioorthogonal reaction is typically a click reaction which is feasible under biologically friendly conditions [34,35,37,38,39]. Most of the requirements for a bioorthogonal reaction are possessed by the azido group, and therefore it has been extensively employed as click functionality in the literature due to its stability and inertness towards other functional groups present in biological systems [31]. Furthermore, the small size of the azido group is particularly interesting because its conjugation to a biovector does not significantly impact the bioactivity of the resulting conjugate [31,40].

### 2.1. The Staudinger Ligation 

This bioorthogonal reaction was discovered by Staudinger and Meyer in 1919 (Table 1) [41,42]. It involves the formation of an aza-ylide via coupling of a triarylphosphine and an azide. The reaction can occur in a living organism and is highly selective because both reagents are abiotic and don’t react with biogenic functionalities of biomolecules. However, the main shortcoming of this reaction is the lack of stability of the aza-ylide product in water [43,44]. Therefore, Saxon and coworkers proposed stabilization of the product of the Staudinger ligation by an intramolecular cyclization. They used an electrophilic trap to capture the nucleophilic aza-ylide by adding an ester group on one of the aryl groups of the phosphine reagent at the ortho position of the phosphorus atom. The reaction led to a stable product via an amide bond formation [43]. Later on, Saxon and coworkers developed another method, dubbed the “traceless” Staudinger ligation, allowing obtention of the amide bond between the two reagents without the intervention of the triarylphosphine group [45].

The Staudinger ligation has been extensively studied in the context of in vitro and in vivo applications [46,47]. However, despite all the efforts to optimize the reaction, it still suffers from some limitations prohibiting its application to in vivo studies. In fact, oxidation of the phosphine and slow kinetics (k ∼ 10^−3^ M^−1^s^−1^) are the main obstacles hampering successful application of the Staudinger reaction in a living system [44,48]. Consequently, further investigations in the realm of bioorthogonal chemistry have been pursued to find click reactions with better chemical properties. 

### 2.2. Copper-Catalyzed [3 + 2] Azide–Alkyne Cycloaddition (CuAAC) 

The azide-alkyne cycloaddition was originally introduced by Huisgen. This classical [3 + 2] cycloaddition is characterized by relatively slow kinetic, thus it is not compatible with pretargeting in biological environments. In 2002, the copper catalyzed azide-alkyne cycloaddition (CuAAC) was described by Sharpless and Mendal [49,50]. The use of Cu(I), as a catalyst increased the second order reaction rate by seven orders of magnitude compared to the uncatalyzed reaction [37]. The kinetics of CuAAC (10 M^−1^ s^−1^ in presence of 20 µM of Cu(I)) are 1000-fold faster than the Staudinger ligation (Table 1) [51]. Moreover, the CuAAC fulfills all the conditions to be classified as a click reaction. The small size of the two functional groups involved, namely azide and alkyne, enabled the incorporation of the click handles into biomolecules without disrupting their biochemical properties. Thus, CuAAC has been applied to the labeling of peptides and proteins [52]. Nevertheless, biological applications of CuAAC has been hampered by the cytotoxicity of copper, which is known to participate in the generation of reactive oxygen species (ROS) [53]. In fact, in 2009, Bertozzi et al. reported that mammalian cells could survive only one hour exposure to low concentrations of copper (lower than 500 μM) [37]. Therefore, to improve the biocompatibility of the reaction, water-soluble Cu(I) ligands were developed to stabilize the metal and prevent the release of toxic copper ions [54,55]. Another approach is to perform the [3 + 2] azide–alkyne cycloaddition with a strained alkyne, which avoids the need of copper catalyst.

### 2.3. Strain-Promoted [3 + 2] Azide–Alkyne Cycloaddition (SPAAC) 

The strain-promoted [3 + 2] azide–alkyne cycloaddition (SPAAC) was introduced in 2004 by Bertozzi et al. [56]. The main advantage of SPAAC in comparison to CuAAC is that the reaction occurs under physiological conditions and without a catalyst. However, the reaction kinetics (1.2–2.4 × 10^−4^ M^−1^ s^−1^) are slower than CuAAC and comparable to those of the Staudinger ligation [57]. Nevertheless, the slow kinetics of the reaction were improved by structural modifications of the alkyne, such as fluorination and sp^2^-hybridization of ring atoms [57,58]. By reaching 0.1 M^−1^ s^−1^, Bertozzi et al. were able to accelerate by 60-fold the reaction kinetics (Table 1) [59,60]. SPAAC has been tested in different systems including the labeling of glycol chitosan nanoparticles with copper-64 or pretargeted radioimmunotherapy (PRIT) of non-Hodgkin lymphoma [61,62,63]. Although SPAAC does not require toxic catalyst and exhibits kinetics 100-fold faster than the Staudinger ligation, it has been shown not to be perfectly bioorthogonal. The cyclooctyne ring can potentially react with nucleophiles present in living systems. Therefore, new type of bioorthogonal reaction offering higher kinetics and better chemoselectivity are needed.

### 2.4. Inverse Electron-Demand Diels-Alder (IEDDA)

IEDDA was introduced in 2008 by Blackman et al. as the fastest bioorthogonal reaction [66]. The reaction occurs between a diene, such as 1,2,4,5-tetrazine (Tz), and a dienophile. Contrary to the electron-demand Diels-Alder reaction, an electron-rich dienophile reacts with an electron-poor diene in IEDDA. According to the frontier molecular orbital theory (FMO), the fast reaction kinetics of IEDDA are due to the low energy gap between the highest occupied molecular orbital (HOMO) and the lowest unoccupied molecular orbital (LUMO) of the dienophile and diene, respectively [48]. The reaction can be set in organic solvents, water, as well as biological media, and does not require activation by a catalyst [66]. Moreover, reactants can be used at very low concentration for their conjugation to large biomolecules due to the high chemoselectivity of the reaction [48].

*Trans*-cyclooctene (TCO) is the most commonly used dienophile due to its high reactivity towards diene. In fact, TCO is seven-fold more reactive than the *cis*-cyclooctene in IEDDA reaction. Tetrazines are generally employed as dienes, and the IEDDA is sometimes called tetrazine ligation [67]. The Tz and TCO pair shows very high reaction specificity, and they are not reactive toward thiols, amines and other potential nucleophiles present in the biological system. This irreversible process leads to the release of N_2_ gas, as the only side product during the reaction [68]. The reaction between Tz and a dienophile can be monitored spectroscopically by following the disappearance of the absorption band between 510 and 550 nm [69]. This method was used by Sauer and coworkers to perform kinetic studies proving that this reaction is incredibly fast [70]. Its exceptional fast kinetics are reported between 1 and 10^6^ M^−1^ s^−1^. Due to all those reasons, IEDDA is so far the most efficient bioorthogonal reaction reported in the literature [71]. 

## 3. Dienes

**Figure 2:**
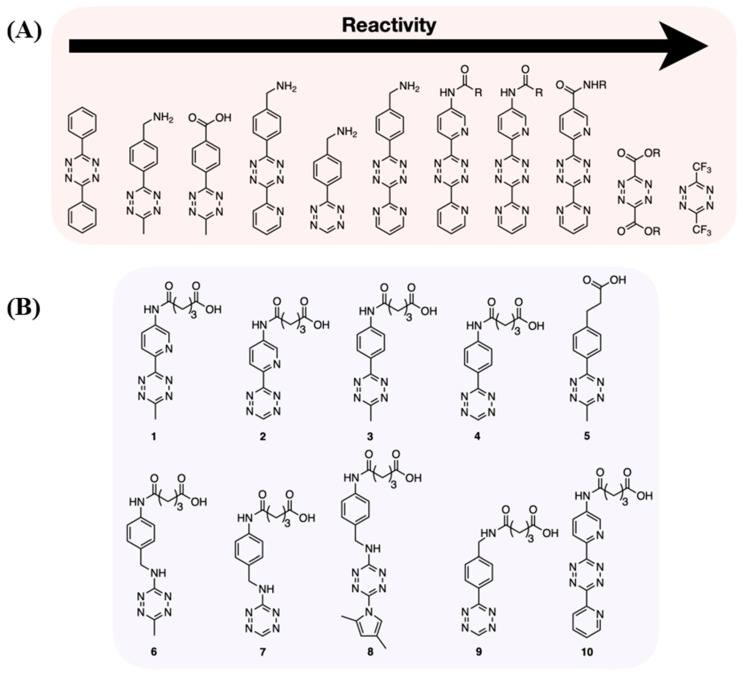
(**A**) Reactivity of different Tz-scaffolds [14] and (**B**) chemical structures of the newly synthesized tetrazine (**1**–**8**) and common tetrazines (**9**, **10**) [72].

Dienes are one of the two click functionalities required for the IEDDA reaction, and their stability is a key issue for in vivo pretargeting. Tetrazines (Tz) are the most commonly used dienes for IEDDA. The reactivity of Tz is influenced by the substitutions performed on the tetrazine backbone (Figure 2A) [14]. Installation of an electron-withdrawing group (i.e., aryl group) on Tz lowers the LUMO energy and leads to high reactivity. Unfortunately, the reactivity of tetrazines is inversely correlated with their stability. In 2016, Maggi et al. investigated the stability of a series of 10 tetrazines to understand the effects of different substituents on the tetrazine core (Figure 2B) [72]. In general, *C*1-monoaryl-substituted tetrazines (**2**, **4**, **8**, **9**) were found to easily decompose under biological conditions (PBS or FBS). The stability can be restored by introduction of a methyl group at the *C*4-position (**1**, **3**, **5**). Installation of electron-donating group at either the *C*1 or both *C*1/*C*4 positions stabilized the tetrazine core but also reduced the reactivity (**6**, **7**). However, the reactivity between Tz and TCO can be enhanced by increasing the polarity of the solvent or the temperature [73]. The di-aryl substituted tetrazine, 3,6-di-(2-pyridyl)-1,2,4,5-tetrazine **10**, is the diene that exhibited the worst stability, as well as the best reactivity among all tetrazines tested. Recently, Steen et al. developed a library of 45 tetrazines and compared their lipophilicity (clogD_7.4_), topological polar surface areas (TPSAs) and in vivo IEDDA reactivity [74]. A strong correlation was noticed between the tetrazine ligation efficiency and the lipophilicity of the Tz. Negative clogD_7.4_ values of −3.0 or lower great improve IEDDA efficiency. However, no correlation was observed between TPSA and the click reactivity. They also reported that a very high second-order rate constant (>50,000 M^−1^ s^−1^) is important to achieve efficient in vivo IEDDA reaction.

## 4. Dienophiles

**Figure 3:**
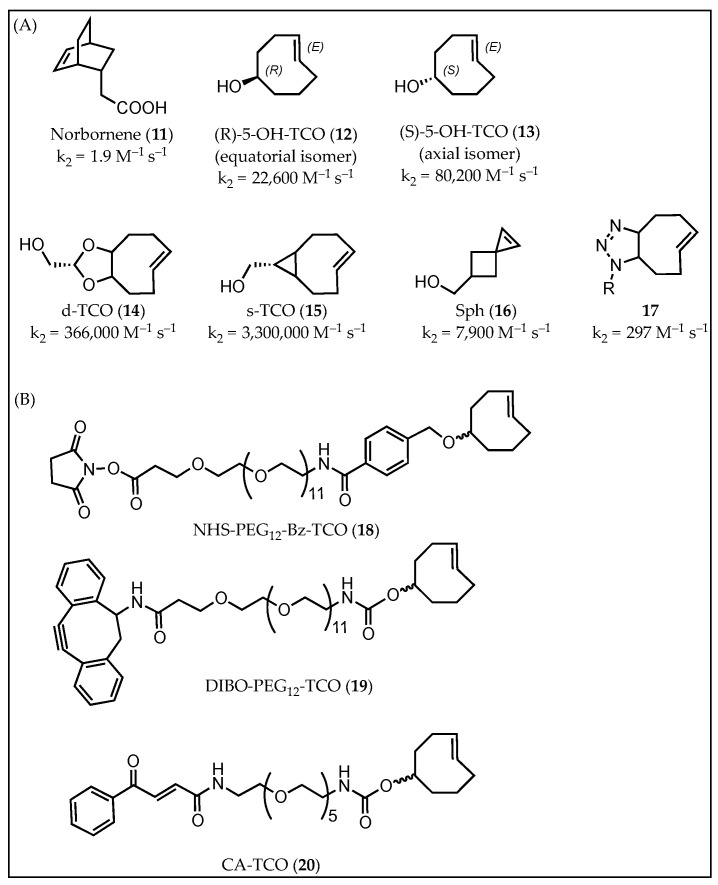
(**A**) Represented dienophiles for IEDDA reaction: **11** [75], **12**–**15** [77], **16** [78], **17** [80]. (**B**) Modified dienophiles for antibody-based pretargeting studies.

The reaction of an electron-rich dienophile and an electron-poor diene is the basis of the IEDDA reaction. Norbornene (**11**) is one of the earliest dienophiles applied for pretargeting studies (Figure 3A). Devaraj et al. demonstrated that tetrazine-substituted imaging probe specifically conjugated to a norbornene-modified HER2-antibody in the presence of live cells and serum [75]. However, the slow kinetics of this reaction (*k*_2_ = 1.9 M^−1^ s^−1^) prevented further applications. IEDDA kinetics are highly dependent on the reaction substrates, and therefore the activity of various dienophiles has been extensively investigated in recent years. Since the 1990s, Sauer’s group and other research groups have examined the reactivity of substituted dienophiles and several principal rules have been summarized: (1) dienophiles with electron-rich substituents are favorable for fast kinetics; (2) strained dienophiles are more reactive, and (3) an increase of the steric effect hampers reactivity. Currently, the most widely used dienophile for IEDDA-based pretargeting studies is *trans*-cyclooctene (TCO). TCO is usually functionalized with a hydroxyl group at the 5-position, known as 5-hydroxy-*trans*-cyclooctene (5-OH-TCO), for further conjugation. There are two stereoisomers of 5-OH-TCO (**12** and **13**), possessing different reactivity. The axial isomer **13** was found to be more reactive than the corresponding equatorial isomer **12**. However, despite the fast kinetics observed with these TCOs, the TCO-tag was found to be partially deactivated in vivo through isomerization to a slow-reactive *cis*-cyclooctene (CCO) [76]. Elaboration of TCO with a short linker was found to extent the half-life of the *trans*-configuration of TCO. In addition to TCO, other dienophiles with higher reactivity (**14** and **15**) [77] or better stability (**16**) [78] have been reported. Furthermore, a bifunctional dienophile (*trans,trans*-1,5-cyclooctadiene, ((*E,E*)-COD)), allowing double click reactions was reported by Leeper’s group. COD is capable of undergoing a (3+2) cycloaddition with 1,3-dipoles to generate triazoline-TCOs (**17**), followed by an IEDDA reaction with tetrazine moieties [79]. The chemistry allowed less-tedious chemical modification of TCO and was exploited by Longo et al. to prepare several TCO derivatives for live-cell fluorescent imaging. However, the kinetics of **17** were too slow for an in vivo pretargeting study [80]. So far, and to the best of our knowledge, no other dienophiles than 5-OH-TCO have been successfully advanced to in vivo pretargeting studies. 

5-OH-TCO was first reported in antibody-based pretargeting studies by Robillard et al. [22]. In this study, (R)-5-OH-TCO was functionalized with a benzoic acid-oligoethylene glycol hybrid linker (NHS-PEG_12_-Bz-TCO, **18**) and conjugated to the anti-TAG72 antibody CC49 (Figure 3B). The resulting TCO-CC49 had a second order rate kinetic constant of 13′090 M^−1^s^−1^ in PBS buffer. It was demonstrated that TCO-CC49 was still reactive after 24 h blood circulation, thus indicating good in vivo stability. In 2016, Cook et al. introduced the activated cyclooctyne DIBO onto 5-OH-TCO (DIBO-PEG_12_-TCO) for site-specific labeling of antibody. Four azido functional groups were installed on the heavy chain of the ^ss^huA33 antibody via a modular chemoenzymatic strategy with β-1,4-galactosidase and galactosyltransferase (Gal-T(Y289L)). Then, the azido-antibody was conjugated to DIBO-PEG_12_-TCO (**19**) according to a SPAAC reaction to adapt the antibody to IEDDA-mediated pretargeting studies [18]. MALDI-TOF mass spectrometry and denaturing SDS-PAGE analyses showed that approximately 2.4 TCO groups were attached on the heavy chain of the antibody, while immunoreactivity of the conjugate was almost not influenced by the modifications (94%). Another site-specific labeling method based on reaction between cysteine and carbonylacrylic group was recently reported [81]. 5-OH-TCO was functionalized with a carbonylacrylic group (**20**) and conjugated to THIOMAB LC-V205C, a modified anti-HER2 antibody with cysteines in the light chain. LC-mass spectrometry analysis of THIOMAB LC-V205C-TCO revealed that the immunoconjugate was modified with two TCO/mAb. The reaction kinetics of THIOMAB LC-V205C-TCO were investigated by performing the click reaction with [^111^In]In-DOTA-Tz. It resulted in ~92% conversion yield, but the immunoreactivity of the conjugate was not reported.

## 5. Applications

### 5.1. Radiolabeling of Monoclonal Antibodies (mAbs) with Short-Lived Radionuclides and Pretargeting

Radiolabeled mAbs are widely used in preclinical and clinical positron emission tomography (PET) imaging studies due to their high selectivity for specific antigens expressed at the surface of tumor cells [82,83,84,85]. However, the long biological half-life of mAbs calls for labeling with long-lived radionuclides to warrant sufficient accumulation of radioactivity at the tumor sites. This approach is unfortunately limited by the long circulation of the radiolabeled molecule in the blood and the radiotoxicity induced to nontargeted organs [86]. To overcome this limitation, Ruivo and coworkers made use of the pretargeting method to combine the specificity of mAbs with the fast pharmacokinetics of radiolabeled small molecules. IEDDA-mediated pretargeting gave them the opportunity to perform PET imaging with mAbs and short-lived radionuclides. In 2015, Houghton et al. applied a similar strategy to improve the prognosis of pancreatic ductal adenocarcinoma (PDAC) [87]. In fact, carbohydrate antigen 19.9 (CA19.9) is highly expressed in PDAC and can be selected as molecular target for PET imaging of pancreatic cancer. However, its secretion in blood results in an increase of circulating CA19.9-targeting radiotracer and decrease in tumor uptake. Therefore, the authors proposed a pretargeting strategy based on TCO-modified monoclonal antibody targeting CA19.9 (5B1-TCO) and copper-64 labeled tetrazines ([^64^Cu]Cu-NOTA-Tz and [^64^Cu]Cu-NOTA-PEG_7_-Tz). They evaluated the optimal time interval (48, 72 and 120 h) between the two injections and compared the pharmacokinetics of the two radiolabeled tetrazines. It was found that 72 h gave the best balance between blood clearance and chemical stability of TCO. Furthermore, the addition of the PEG_7_ linker improved hydrophilicity of the ^64^Cu-labeled tetrazine and reduced accumulation in the gastrointestinal tract. Later, they reported a fluorine-18 labeled tetrazine (Tz-PEG_11_-Al [^18^F]-NOTA), which could be obtained in mild reaction conditions to avoid alkaline degradation of the tetrazine [88]. Imaging of athymic mice bearing subcutaneous CA19.9-expressing BxPC3 xenografts was performed with Tz-PEG_11_-Al[^18^F]-NOTA 72 h after injection of 5B1-TCO. Ex vivo biodistribution revealed increase tumor uptake overtime, which was confirmed by PET imaging data. Several other ^18^F-labeled tetrazines have been developed due to the attractive decay properties (*t*_1/2_ = 109.8 min) of fluorine-18 for PET imaging [86]. Keinänen et al. described a method for the preparation of a ^18^F-labeled glycosylated tetrazine by oxime ligation between 5-[^18^F]fluoro-5-deoxyribose and an aminooxy functionalized tetrazine [89]. Addition of the sugar moiety reduced the lipophilicity of the labeled tetrazine. In vitro stability of the radiofluorinated tetrazine in PBS buffer and mouse plasma showed no degradation in PBS over 6 h, while 90% of the compound remained intact in plasma. Ex vivo biodistribution studies revealed low level of in vivo defluorination and rapid elimination of the radiotracer via the bladder, confirming the potential of this ^18^F-labeled tetrazine for pretargeting purposes. 

Zeglis and coworkers reported several IEDDA-mediated pretargeting studies using monoclonal antibodies functionalized with a TCO and a radiolabeled tetrazine [90,91,92,93,94]. They recently described an efficient radiosynthetic approach based on IEDDA to prepare actinium-225 radioimmunoconjugates [90], while previous methods presented major limitations, such as radiolytic degradation or the need of a high number of chelate per antibody (~10), which could potentially affect the radioimmunoreactivity of the conjugate. Meyer et al. reported a masking agent to neutralize the free circulating mAb-TCO before the injection of the radiolabeled tetrazine. The main objective was to increase the target-to-background ratios without affecting tumoral uptake. It was shown that the quality of the PET images was significantly improved for the mice who received the masking agent in comparison to the control animals [91]. Then, Cook and coworkers investigated the possibility to increase the number of TCO groups per antibody. They synthesized a TCO-bearing scaffold based on a disulfide-core poly(amidoamine) (PAMAM) dendrimer, which was regioselectively attached to the ^ss^huA33 antibody (^ss^huA33-DEN-TCO). Biodistribution and microPET showed that the tumor uptake after injection of [^64^Cu]Cu-SarAr-Tz was two-fold higher in mice treated with ^ss^huA33-DEN-TCO compared to the animals treated with ^ss^huA33-PEG_12_-TCO [95].

**Figure 4:**
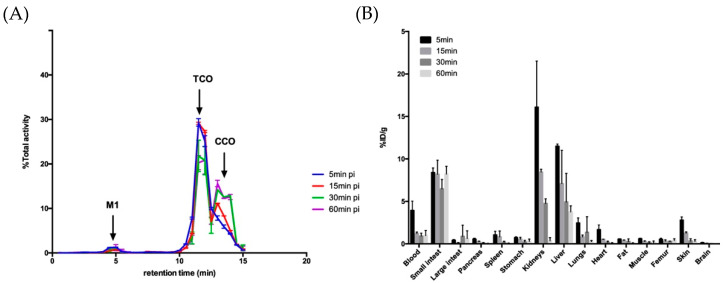
(**A**) In vivo plasma metabolites and (**B**) biodistribution of [^18^F]F-MICA-205 in healthy Balb/C mice at 1 h post injection [86].

**Figure 5:**
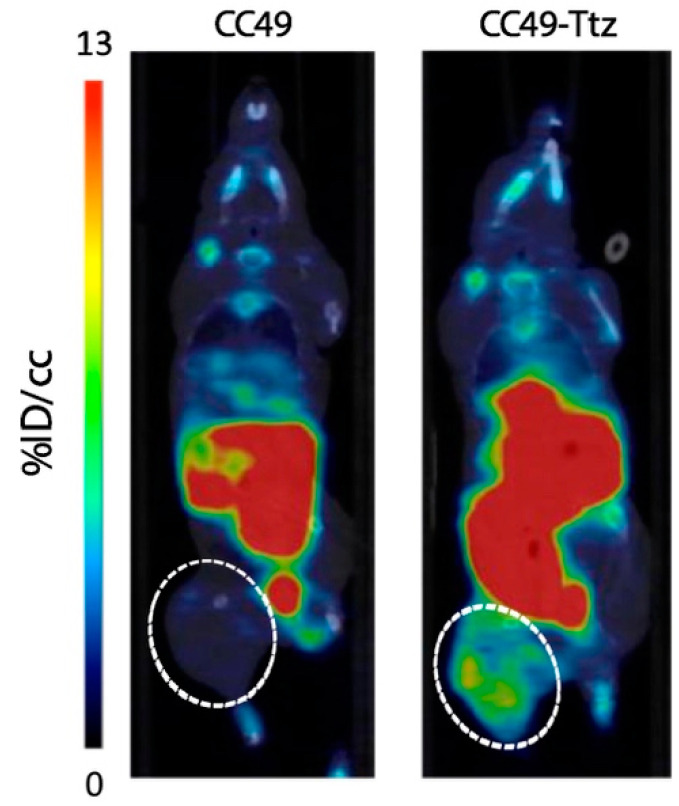
Representative microPET images of LS174T tumor-bearing mice injected with [^18^F]F-MICA-205 24 h after the injection of CC49 (left) or CC49-Tz (right). Static images were acquired 1 h after administration of the [^18^F]F-MICA-205. The white dashed line encircles the tumors [86].

In the previous studies, the dienophile was conjugated to the biovector, while the diene was radiolabeled. However, due to the long-term in vivo instability of TCO, Maggi et al. proposed attachment of the tetrazine to the antibody and radiolabeling the TCO moiety [72,76,96]. Radiofluorination of TCO was attempted to obtain [^18^F]F-TCO, but the tracer was rapidly metabolized and a nonspecific accumulation in bones via defluorination was observed. Consequently, [^18^F]F-TCO has not been successfully applied to pretargeting studies [97,98]. In 2017, Billaud et al. performed fluorine-18 labeling of a pegylated TCO for pretargeted immuno-PET imaging. Animals with HER-2 positive xenografts were first injected with a trastuzumab-tetrazine conjugate, followed by the administration 48 to 72 h later of the ^18^F-labeled TCO. The HER-2 overexpressing tumors could clearly be visualized on the PET images, proving that pretargeting immuno-PET imaging can be achieved with this fluorinated TCO analog [99]. Later, Ruivo and coworkers modified the TCO labeling method by using the 1,4,7-triazacyclononane-*N,N’,N″*-triacetic acid (NOTA) chelator. TCO was radiolabeled by complexation of Al[^18^F]F by the NOTA chelator, and [^18^F]F-MICA-205 was evaluated in vivo as a probable counterpart for the IEDDA reaction with a tetrazine-modified CC49 antibody. In vivo stability studies showed that 67.7 ± 0.43% of [^18^F]F-MICA-205 remained intact 15 min after injection (p.i.) of the tracer, whereas it decreased to 51.9 ± 5.16% at 1 h p.i.. Notably, it was identified that the main radiometabolite at the later timepoint was the *cis*-isomer (Figure 4A). However, the *trans* to *cis* isomerization was not considered a major limitation because of the fast kinetics of the IEDDA reaction. Biodistribution of [^18^F]F-MICA-205 in healthy mice showed both a hepatobiliary and a renal excretion due to the hydrophobic character of the tracer. As illustrated in Figure 4B, most of the radioactivity was found in the kidneys, the liver, and the small intestine. The low uptake in bones demonstrated the in vivo stability of the radiotracer. In vivo pretargeting studies were performed in LS174T tumor-bearing mice pretreated with the anti-TAG-72 mAb CC49 or with the same antibody conjugated to a stable methyl-tetrazine. [^18^F]F-MICA-205 was injected and microPET imaging was performed 1 h after administration of the tracer (Figure 5). The tumor uptake in mice pretreated with CC49-Tz (0.67 ± 0.16% ID/g) was significantly higher than the control animals preinjected with CC49 (0.16 ± 0.08% ID/g). It clearly demonstrated that the radiolabeled TCO, [^18^F]F-MICA-205, could be applied to an in vivo pretargeting strategy.

### 5.2. Radiolabeling of Nanoparticles 

**Figure 6:**
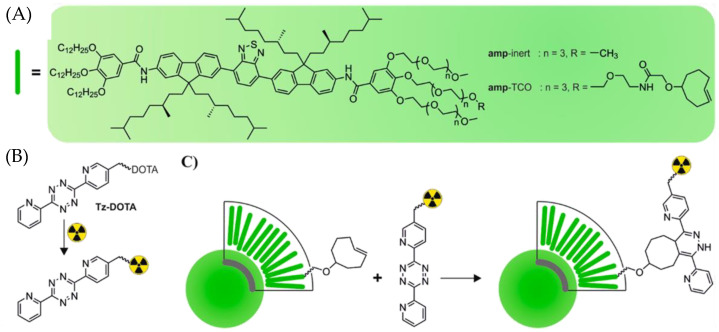
(**A**) Chemical structures of the amp-inert and amp-TCO [102], which self-assemble into SMNPs. (**B**) Two-step radiolabeling strategy by chelation of the radionuclide by Tz-DOTA followed by (**C**) conjugation of labeled Tz-DOTA to amp-TCO [101].

The fast kinetics and chemoselectivity of IEDDA are particularly attractive for the selective labeling of complex molecules such as nanoparticles. Recently, Goos et al. developed nanostars for multimodality molecular imaging and endoradiotherapy [100]. Nanoparticles were complexed with Gd^3+^ for T_1_-weighted magnetic resonance imaging and functionalized with a TCO group, p(TCO-AEA-co-OEGA-co-[Gd^3+^]VDMD, to enable radiolabeling via IEDDA reaction. Tetrazine ligation was performed between p(TCO-AEA-co-OEGA-co-[Gd^3+^]VDMD and [^177^Lu]Lu-Tz-PEG_7_-DOTA to obtain p([^177^Lu]Lu-DPAEA- *co*-OEGA-*co*-[Gd^3+^]VDMD. The ^177^Lu-labeled nanostars showed high uptake in the tumor tissue in comparison to other nanoparticles previously reported in the literature. In 2019, van Onzen and coworkers also used the selectivity of the bioorthogonal reaction between Tz and TCO to radiolabel nanoparticles, which were intrinsically fluorescent, and obtained dual-modality imaging probes (Figure 6) [101]. They incorporated a *trans*-cylooctene moiety to small molecule-based nanoparticles (SMNP) followed by the addition of a tetrazine-DOTA conjugate (Tz-DOTA) previously labeled with indium-111. The dual-modality imaging probe was obtained in over 97% radiochemical yield by reacting [^111^In]In-DOTA-Tz with the cyclooctene unit (amp-TCO) in buffer for 30 min. The authors also compared this radiosynthetic strategy with two conventional labeling approaches where the DOTA chelate was directly attached to the SMNPs (amp-DOTA) or conjugated to the nanoparticles by strain-promoted azide-alkyne cycloaddition. The radiolabeling yield dropped to 45% for [^111^In]In-DOTA-amp, and the radiochemical purity reached 88% after purification of the labeled nanoparticles by size exclusion chromatography. However, no reaction was observed during SPAAC with the azido-modified SMNPs (amp-azide), probably due to steric hindrances. This example clearly demonstrates the advantages of IEDDA over other techniques to radiolabel nanoparticles. 

Recently, Keinänen et al. reported the development of an imaging strategy based on the IEDDA reaction and mesoporous silicon nanoparticles (PSi-NPs) [103]. Mesoporous silicon is an interesting material for targeted drug delivery in nanomedicine due to the biodegradability and nontoxicity of Psi-NPs. TCO-NPs were obtained by SPAAC reaction with TCO-PEG_12_-DBCO after the conjugation of 3-azidopropylamine onto the NPs. IEDDA was carried out with [^18^F]fluorodeoxyribose-tetrazine ([^18^F]FDR-tetrazine). TCO-NPs were administrated intravenously to healthy mice 15 min or 24 h prior to the injection of [^18^F]FDR-tetrazine. A control group was treated only with [^18^F]FDR-tetrazine. PET-CT images revealed that the highest radioactive uptake was found in the spleen for the group treated with TCO-NPs 15 min prior [^18^F]FDR-tetrazine administration, as typically observed for NPs. Authors reported that the click reaction between both moieties, TCO and Tz, was rapid and that 11.0 ± 1.9% ID/g was found in the spleen (vs. 2.8 ± 0.5% ID/g for the control group). However, no click reaction was observed for the group treated with TCO-NPs 24 h before the administration of the radiolabeled Tz. This might be due to the loss of reactivity of the TCO group and conversion to its unreactive *cis*-isomer, or the TCO-NPs were already completely internalized.

### 5.3. Drug to Release

**Figure 7:**
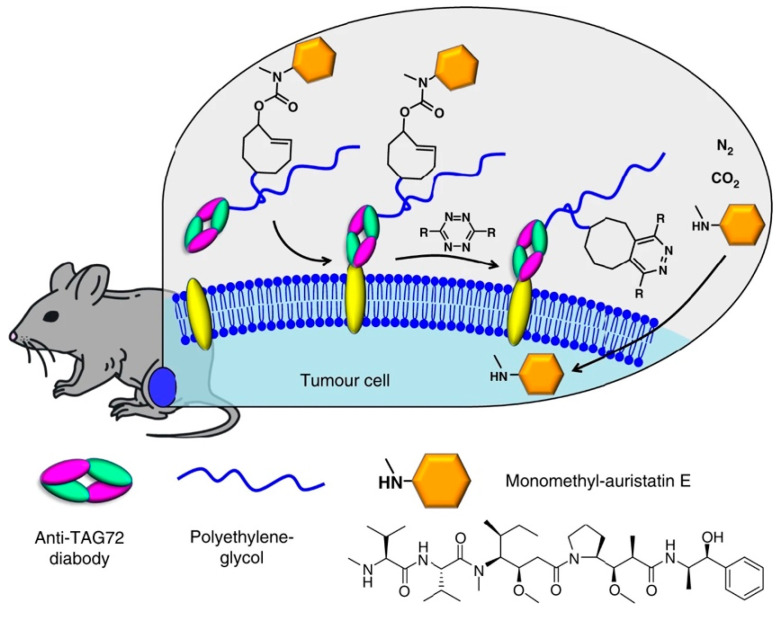
Triggered drug release using “click-to-release” chemistry in vivo: on-tumor liberation of a cell permeable drug (monomethyl auristatin E, MMAE) from a *trans*-cyclooctene-linked ADC following systemic administration of a tetrazine activator [104].

The principle of the pretargeting strategy, consisting of the injection of the targeting vector followed by the administration of an active ingredient interacting solely with the targeting vector, has not exclusively been used for the safe administration of a radioactive substance but also for the site-specific release of a drug. In 2018, Rossin et al. showed that the reaction of a tetrazine with a TCO linked to an antibody-drug conjugate (ADC) bound to a noninternalizing cancer cell receptor could lead to intracellular release of the drug while sparing the surrounding healthy tissues (Figure 7) [104]. In fact, their “click-to-release” strategy was based on previously published results showing the specific cleavage of allylic carbamates from TCO upon reaction with Tz [105,106]. 

As proof-of-concept, the authors selected the tumor-associated glycoprotein-72 (TAG72), as the noninternalizing receptor, and CC49 diabody conjugated to a PEG_24_ linker and coupled to *trans*-cyclooctene bound to a tubulin-binding antimitotic MMAE (tc-ADC) to target TAG72. tc-ADC was compared to vc-ADC containing an enzymatically cleavable valine-citrulline linker and nb-ADC, a nonbinding anti-PSMA containing the TCO linker. To release the drug by the IEDDA reaction, a 3-methyl-6-trimethylene-tetrazine (activator) was employed. This tetrazine is known to be less reactive than the 3,6-bispyridyl-tetrazine previously reported for successful in vivo IEDDA, but it presents better releasing properties. To determine the efficiency of the “click-to-release” strategy, the click reaction was first performed in PBS buffer and in serum. Upon addition of the Tz activator, 90% of the drug was released in PBS buffer within 1 h of incubation at 37 °C, whereas in serum 80% of drug was released in 20 h. Subsequently, in vivo studies in LS174T-tumor bearing mice were carried out after the injection of the activator 48 h after the injection of tc-ADC. Biodistribution was performed at 72 or 96 h post injection of tc-ADC. MMAE release was estimated by mass spectrometry from liver and tumor homogenates as well as plasma. MMAE levels were 100-fold higher in the tumors treated by the subsequent injections of tc-ADC and the activator in comparison to the liver, the plasma or tumors treated solely with tc-ADC. Then, therapy studies were performed to compare the efficacy of tc-ADC + 3-methyl-6-trimethylene-tetrazine and vc-ADC. It was found that the click-to-release approach had a strong and durable response in OVCAR-3 xenografts without any signs of toxicity up to four months after treatment, while vc-ADC was not effective in this tumor model.

Recently, Li et al. used the same technique to investigate localized molecular imaging with tumor specificity and spatiotemporal precision [107]. In fact, fluorescence imaging showed very promising results in tumor diagnosis, but due to poor tissue penetration the results showed modest outcome. Therefore, in this study, single-walled carbon nanotubes (SWCNTs) attached to Tz (Tz@SWCNTs) were used as delivery vehicles to increase accumulation in tumors via enhanced permeability and retention effects (EPR). For the second reagent of the IEDDA reaction, TCO was coupled to hemicyanine to yield (tHCA). tHCA is a fluorogenic near infrared probe that becomes fluorescent upon activation. The authors reported the ability of tHCA to diffuse deeply into the cancer tissue and to lead to tumor-specific imaging upon click reaction with Tz@SWCNTs. Thus, this strategy allowed deep tissue penetration via SWCNTs, and high signal-to-noise ratio by the activation of tHCA. This method enabled specific, nondestructive and real-time imaging. Then, they investigated pretargeted fluorogenic imaging in live cells, as well as in vivo. For their in vitro studies, MCF-7 cells were incubated with Tz@SWCNTs for 6 h before the addition of tHCA. The first fluorescent signal was reported after 5 min and its intensity increased over 30 min. In vivo studies were performed in CT26 tumors bearing BALB/c mice. For the group of mice intended for pretargeted imaging, mice were injected intravenously with Tz@SWCNTs and 2 h later with tHCA. The images showed a signal emerging from the tumor at 3 h post injection of tHCA. The intensity of the signal continued to increase up to 24 h after administration of tHCA. Based on their results, the authors identified a method for real-time fluorescence imaging with spatiotemporal control.

### 5.4. Activatable Fluorescence Probes

Fluorescence imaging of live cells with caged fluorescent probes is a powerful technique to study dynamic cellular processes and events [108]. In 2010, Weissleder et al. first reported that Tz-BODIPY conjugates (e.g., tetrazine-BODIPYFL) exhibited strongly reduced fluorescence compared to their parent BODIPYs [109]. The quenching mechanism was suggested to be the result of a Förster resonance energy transfer (FRET) process between the electron-poor Tz and the fluorophore. Upon reaction with a dienophile, such as TCO, the fluorescence could be restored in the order of 15 to 20-fold. For live-cell study, the authors sequentially treated PtK2 kidney cells with taxol-TCO and tetrazine-BODIPYFL. In vitro, the IEDDA reaction was successfully demonstrated by clear visualization of the cellular tubule networks. Recently, the same authors developed another series of “superbright turn-on probes” (e.g., mTz-BODIPY, HELIOS) based on a through-bond energy transfer (TBET) process in which the Tz, acting as a quencher was attached to the fluorophore via a rigid linker [110,111]. Fluorescence increase in the order of 10^3^ to 10^4^ was observed after TCO-activation of the probes. From a mechanistic point of view, the TBET-based quenching process can be applied to any type of fluorophore, and Kele’s group reported Tz-caged probes based on various fluorophores, including phenoxazine, coumarin, siliconrhodamine and rhodamine analogs [112,113,114,115]. The feasibility of applying the probes to cell-based imaging has also been demonstrated by confocal microscopy. Later, Kele et al. developed new IEDDA-activatable fluorogenic photocages based on a vinylene linked coumarinyl-Tz, which could be activated through the IEDDA reaction with a strained alkyne. Live-cell photouncaging was demonstrated by pretargeting the cells with an alkyne (TPP-BCN) followed by the addition of the fluorogenic rhodol-coumarinyl-Tz agent, resulting in fluorescence signal located in cell mitochondria [116].

### 5.5. Photodynamic Therapy

Photodynamic therapy (PDT) is a clinically approved medical treatment that involves a photosensitizing molecule and a light source to destroy malignant cells. The treatment depends on the direct or indirect generation of cytotoxic singlet oxygen (^1^O_2_) or other ROSs (e.g., peroxide radicals, hydroxyl radicals) under exposure of the photosensitizer (PS) to light. Because the lifetime and diffusion distance of ^1^O_2_ is short, specific delivery of PS at the target site is essential. Recently, Renard et al. took advantage of the IEDDA reaction to simultaneously label an EGFR-targeted VHH antibody with a complexed radionuclide (^111^In-DTPA) and a PS (IRDye700DX) [117]. Micro-SPECT and near infrared fluorescence (NIFR) imaging showed that the tracer specifically accumulated in the tumor. Moreover, the dual-modality VHH exhibited dose-dependent cytotoxicity upon illumination with 60 J/cm^2^ 690 nm light in an in vitro assay. Despite the success of targeted PDT, controllable activation of the PS is still under investigation to improve the effectiveness of PDT. Pioneer work on halogenated BODIPY-Tz by Vázquez’s group demonstrated that the photosensitizer could be turned on and effectively generate ^1^O_2_ through IEDDA reaction [118]. In this study, the tetrazine on the PS not only played the role of a TBET quencher but also a PS inactivator. PS activity could be restored by changing the nature of this quencher via IEDDA reaction. To validate the concept, a 5-vinyl-2′-deoxyuridine (VdU) was incorporated, as a dienophilic activator, into the DNA of HeLa cells. The cells were treated with mTz-2I-BODIPY and irradiated with a light at 525 nm for ^1^O_2_ generation. The DNA-targeted PS activation proved to be successful because the product of the IEDDA reaction led to significant phototoxicity in HeLa cells. They concluded that the IEDDA-based activatable photodynamic strategies could be a useful tool for PDT. Next, Vázquez and coworkers developed a bioorthogonal turn-on peptide PS (Tz-C(2I-BODIPY)-PEPTIDE) [119]. Unlike the previous BODIPY/Tz photosensitizer, the tetrazine and BODIPY moieties were separately integrated into a cell membrane-targeted peptide. In vitro IEDDA reaction followed by proper irradiation led to significant suppression of the HeLa cell viability, showing that the new PS/quencher pair was successfully turned on by TCO treatment. Another example of IEDDA reaction-mediated activatable PS was reported by Dong et al. [120]. They used a tumor pH-responsive polymer containing a tetrazine, which formed unreactive micelles at neutral pH. However, micelles disassembled under the acidic tumor microenvironment (pH 6.5), leading to the activation of the caged PS. The authors demonstrated that the Tz-polymers were released at pH 6.5 and activated the CyPVE (a vinyl-ether-caged fluorogenic PS) through the IEDDA reaction, resulting in the photodynamic cytotoxicity of 4T1 cells. On the contrary, no cytotoxicity was observed at pH 7.4 because the Tz-micelles were stable under neutral conditions. In vivo PDT studies showed that 4T1 tumor xenografts shrunk after 12 days of treatment by the Tz micelles/CyPVE. The successful example of the IEDDA reaction in the context of the acidic tumor microenvironment may provide a general strategy for bioorthogonal prodrug activation.

## 6. Conclusions

To summarize, bioorthogonal reactions have been widely studied over the last few decades for their ability to potentially overcome the limitations in RIT. Although, several click reactions have been introduced, the IEDDA discovered in 2008 by Blackman et al., has been a game changer due to its fast kinetics, high selectivity and biocompatibility. Nowadays, most biological studies involving a bioorthogonal reaction are based on the IEDDA. However, even if some dienes and dienophiles reagents are commonly used, optimization of their chemical properties is still required. Indeed, the in vivo stability of TCO can be compromised in acidic conditions or by the presence of copper ions and nucleophiles in the biological system. Similarly, an equilibrium between reactivity and stability has to be found for the tetrazine. To overcome these limitations, various dienes and dienophiles have been synthesized to optimize the efficiency of the click reaction. For instance, norbornene derivatives or strained cyclopentene have been developed to replace TCO.

Many preclinical studies with TCO or Tz-modified antibodies have shown encouraging results. However, in vivo IEDDA applications with smaller biomolecules, such as peptides, have been barely investigated. Furthermore, implementation of such strategy into clinic is a logistic challenge, considering the number and time between the injections. Additionally, clinical deployment of IEDDA will only be effective for imaging and therapy if it remains affordable. To conclude, IEDDA is a valuable tool for biomedical research to overcome current limitations encountered with direct targeting. However, despite promising preclinical results, further optimization is likely required for the clinical translation of this novel targeting approach.

## Figures and Tables

**Table 1:** Chemical characteristics of the bioorthogonal reactions covered in this review.

Bioorthogonal Reaction	Reaction Scheme	*k* (M^−1^ s^−1^)	Advantages	Drawbacks
Staudinger ligation	A: Staudinger reaction 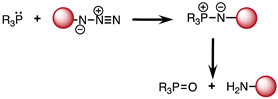	∼10^−3^ [64]	- Abiotic starting materials	- Slow kinetics - Low stability of Aza-ylide
	B: Staudinger ligation 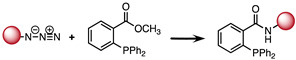	10^−3^	- Stable amide bound formation	- Slow kinetics
	C: “Traceless” Staudinger ligation 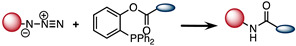	7.7 × 10^−3^ [65]	- Product obtained without the triarylphosphine group	- Slow kinetics
CuAAC	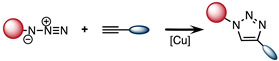	10	- Covalent reaction - Small molecules	- Slow kinetics - Cytotoxicity caused by copper ions
SPAAC	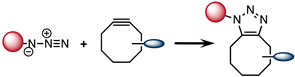	0.1	- Free of catalyst	- Slow kinetics - Reactivity with thiols
IEDDA	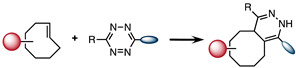	1–10^6^	- Very high second order reaction rate constant - Irreversible reaction - Fully bioorthogonal - Stable product	- Sensitivity of TCO to acids, thiols, and copper ions - Sensitivity of Tz to bases
